# Immune checkpoint signaling and cancer immunotherapy

**DOI:** 10.1038/s41422-020-0343-4

**Published:** 2020-05-28

**Authors:** Xing He, Chenqi Xu

**Affiliations:** 1 State Key Laboratory of Molecular Biology, Shanghai Science Research Center, CAS Center for Excellence in Molecular Cell Science, Shanghai Institute of Biochemistry and Cell Biology, Chinese Academy of Sciences, University of Chinese Academy of Sciences, Shanghai, 200031 China; 2 School of Life Science and Technology, ShanghaiTech University, Shanghai, China

**Keywords:** Tumour immunology, Cancer therapy

## Abstract

Immune checkpoint blockade therapy has become a major weapon in fighting cancer. Antibody drugs, such as anti-PD-1 and anti-PD-L1, demonstrate obvious advantages such as broad applicability across cancer types and durable clinical response when treatment is effective. However, the overall response rates are still unsatisfying, especially for cancers with low mutational burden. Moreover, adverse effects, such as autoimmune symptoms and tumor hyperprogression, present a significant downside in some clinical applications. These challenges reflect the urgent need to fully understand the basic biology of immune checkpoints. In this review, we discuss regulation of immune checkpoint signaling at multiple levels to provide an overview of our current understanding of checkpoint biology. Topics include the regulation of surface expression levels for known immune checkpoint proteins via surface delivery, internalization, recycling, and degradation. Upon reaching the surface, checkpoints engage in both conventional *trans* and also *cis* interactions with ligands to induce signaling and regulate immune responses. Novel therapeutic strategies targeting these pathways in addition to classical checkpoint blockade have recently emerged and been tested in preclinical models, providing new avenues for developing next-generation immunotherapies.

## Introduction

The tumor microenvironment (TME) is infiltrated with many types of innate and adaptive immune cells whose immune surveillance functions are often suppressed by multiple mechanisms in a context-dependent manner.^1,2^ Signaling suppression and metabolic suppression represent two major causes of immune suppression, and the prior will be discussed here. Signaling suppression is reflected by the ways that tumor cells downregulate the activity of stimulatory immunoreceptors while upregulating the activity of inhibitory immunoreceptors. Using T cells as an example, tumor cells can tune down T cell receptor (TCR)-mediated stimulatory signaling by downregulating surface MHC-I level.^3^ On the other hand, tumor cells can tune up PD-1-mediated inhibitory signaling by upregulating surface PD-L1 level.^4^ The concept that blocking the activation of inhibitory immunoreceptors can reinvigorate antitumor function of immune cells has been demonstrated experimentally and translated to treatment of many types of cancer in the clinic.^5,6^

A number of inhibitory immunoreceptors have been identified and studied in cancer in past decades, including but not limited to PD-1, CTLA-4, LAG3, TIM3, TIGIT and BTLA. They are named as “immune checkpoints” referring to molecules that act as gatekeepers of immune responses. In the evolutionary process, immune checkpoints have co-evolved with stimulatory immunoreceptors and appear as early as in fish.^7^ These receptors often use mono-tyrosine signaling motifs, such as immunoreceptor tyrosine-based inhibitory motif (ITIM) and immunoreceptor tyrosine-based switch motif (ITSM), to deliver inhibitory signals. As surface molecules, their activity can be easily inhibited by blocking antibodies that prevent ligand-receptor engagement. The most successful immune checkpoint blockade therapy is anti-PD-1/PD-L1 therapy that has been approved to treat a wide variety of cancer types, such as blood, skin, lung, liver, bladder and kidney cancers.^6^ Immune checkpoint blockade therapy often leads to more durable response than chemo or targeted therapies, perhaps reflecting the memory feature of the immune system. However, as clinical data accumulates worldwide, drawbacks and side effects have begun to be revealed. The major bottleneck of immune checkpoint blockade therapy is its low response rate in most cancers, with a range of 10%–30%.^6^ For some major cancer types such as colorectal cancer with microsatellite stability, anti-PD-1/PD-L1 therapy shows nearly no effect.^8^ Mechanisms of non-responsiveness have been extensively studied, and many factors have been found to be relevant, such as tumor mutational burden, PD-L1 expression level, IFN signaling and MHC-I loss.^9–12^ However, biomarkers that faithfully predict efficacy are still lacking. Better understanding of checkpoint biology is therefore urgently needed to design next-generation therapies and to improve clinical protocols of current therapies.

In recent years, many biochemical and biophysical studies have revealed sophisticated regulation of checkpoint surface expression. Upon ligand engagement, different checkpoints show distinct signaling mechanisms to suppress antitumor immunity. Here we review these fundamental discoveries and highlight new targeting strategies with potential for clinical translation.

## Surface level regulation of immune checkpoints

**Fig. 1:**
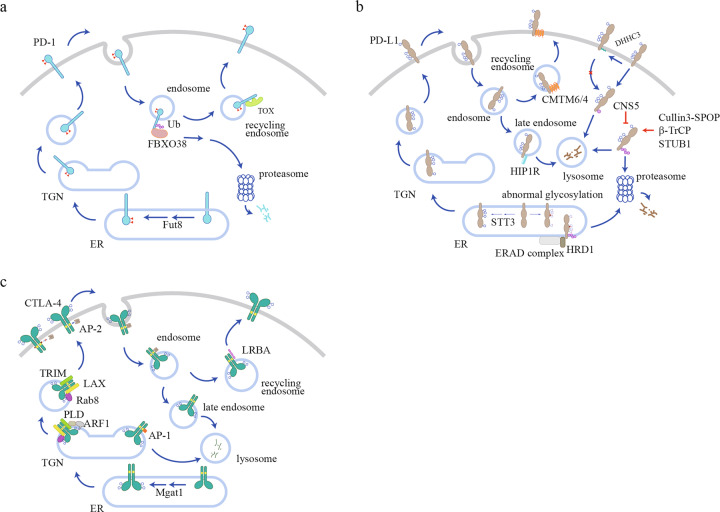
Regulation of surface expression of PD-1, PD-L1 and CTLA-4. **a** Fut8-mediated core fucosylation pathway is required for PD-1 surface expression. Internalized PD-1 is ubiquitinated by FBXO38 for proteasomal degradation and can also be recycled to surface with the help of TOX in liver cancer microenvironment. **b** STT3-catalyzed N-glycosylation stabilizes PD-L1 surface expression. P-S195-induced abnormally glycosylation of PD-L1 causes ERAD. Internalized PD-L1 is either sorted to the lysosome by HIP1R for degradation or recycled to the cell surface with the help of CMTM6/4. PD-L1 is ubiquitinated by different E3 ligases (HRD1, Cullin3-SPOP, β-TrCP and STUB1) under different contexts, and deubiquitinated by CNS5. Palmitoylation of PD-L1 by DHHC3 suppresses its mono-ubiquitination and lysosomal degradation. **c** Mgat1 mediates CTLA-4 N-glycosylation and surface retention. Trafficking of CTLA-4 to the cell surface relies on the TRIM/LAX/Rab8 complex and PLD/ARF1-dependent exocytosis. Rapid CTLA-4 internalization is mediated by AP-2 binding to the unphosphorylated YVKM motif. Internalized CTLA-4 is either degraded in the lysosome or recycled to cell surface by LRBA. CTLA-4 in TGN can also be delivered to the lysosome for degradation through AP-1 binding.

High surface level of checkpoints is a hallmark of TME, but the underlying mechanisms are poorly understood. As membrane proteins, immune checkpoints are expressed in the endoplasmic reticulum (ER) and then delivered to cell surface to exert their inhibitory functions, which involves sequential transportation through Golgi apparatus and secretory vesicle by the protein-sorting system. During surface delivery, glycosylation serves as a quality control to ensure only mature and functional immune checkpoints are delivered to cell surface.^13,14^ After reaching the cell surface, immune checkpoints are subjected to internalization and recycling, which offer a rapid regulatory pathway to modulate their surface levels.^15,16^ Ubiquitination-mediated protein degradation is another crucial mechanism to control protein level and immune checkpoints can be ubiquitinated and sorted to proteasome or lysosome for degradation. These cellular processes together determine surface level of immune checkpoints to shape cell signaling (Fig. 1).

### PD-1 regulations

Human PD-1 contains four N-linked glycosylation sites at its extracellular IgV domain: N49, N58 (N54 in mouse PD-1), N74, and N116. The structure of PD-1 shows that the glycan at N58 consists of two N’acetylglucosamines and one fucose.^17^ Furthermore, whole genome CRISPR screening identified that the core fucosylation pathway directly regulates PD-1 surface level.^18^ Mass spectrometry analysis showed that all four N-glycosylation sites had core fucose modification. Mutation of N49 or N74 caused dramatic decrease of surface PD-1 level. Genetic ablation of the core fucosyltransferase 8 (Fut8) reduced PD-1 surface level and enhanced T cell activation.^18^ How glycosylation regulates PD-1 expression level is still unknown. Sugar groups might regulate PD-1 folding and thus affect the quality control process at the ER. Another possible role of glycosylation is the regulation of ligand binding.^19^ Different microenvironment cues such as hypoxia^20^ and nutrient stress^21^ might cause different glycosylation patterns of PD-1, which can in turn affect PD-1 function. More mass spectrometry studies are warranted in the future to understand the “sugar code” of PD-1 and its functional meaning in specific disease contexts.

Internalization of surface PD-1 has been observed by fluorescence imaging^22^ but it is still unknown whether conventional clathrin-mediated endocytosis is involved in PD-1 internalization. Internalized PD-1 molecules can either recycle back to the cell surface or become ubiquitinated and sorted to proteasome for degradation (Fig. 1a). In liver cancer infiltrating CD8^+^ T cells, the thymocyte selection-associated high mobility group box protein (Tox), a master transcription factor of T cell exhaustion,^23^ was shown to exert a nonconventional function of binding with PD-1 in the cytoplasm and facilitating PD-1 recycling.^24^ Whether Tox also regulates PD-1 recycling in other contexts has not been tested. A specific E3 ubiquitin ligase of PD-1, named F-box protein 38 (FBXO38), has been identified and validated by biochemical and animal experiments. As a part of the Skp, Cullin, F-box containing complex (SCF complex), FBXO38 can mediate K48 polyubiquitination at a conserved lysine site (K233 in human PD-1). Interestingly, polyubiquitinated PD-1 is sorted to the proteasome rather than the lysosome for degradation. This is unusual, as most membrane proteins are internalized and degraded via the lysosome. In the TME, the FBXO38-mediated PD-1 degradation pathway is deficient because of the low transcriptional level of FBXO38. TCR signaling without concomitant CD28 signaling was found to be the cause of FBXO38 downregulation.^22^ Persistent exposure to tumor antigen and low CD80/86 expression on tumor cells^25^ therefore could explain the reduced FBXO38 expression in TILs. Furthermore, IL-2, the major growth factor of T cells, can rescue FBXO38 level in tumor-infiltrating T cells via STAT5-mediated transcriptional regulation.^22^ Notably, FBXO38 expression levels in tumor infiltrating lymphocytes (TILs) are even lower than in naïve T cells. How chronic TCR signaling downregulates FBXO38 transcription is still an open question.

Indeed, in general the processes governing internalization and concomitant degradation or recycling of PD-1 molecules remain poorly understood. Several interesting questions warrant further investigation such as whether PD-1 internalization is signaling-dependent and which signal determines the fate of internalized PD-1, as to whether it is delivered to the proteasome for degradation or recycled back to the cell surface for future usage. These questions are better understood for PD-L1 in cancer cells, as described in the following section.

### PD-L1 regulations

PD-L1 (also named as CD127, B7-H1) also contains four N-glycosylation sites: N35, N192 (N191 in mouse PD-L1), N200 (N199 in mouse PD-L1), and N219 (N218 in mouse PD-L1). These modifications are significant for PD-L1 protein stability. STT3 is an ER-associated N-glycosyltransferase that catalyzes the first step of protein N-glycosylation. In cancer stem-like cells, STT3-dependent N-glycosylation stabilized and upregulated PD-L1 level, which was required for epithelial–mesenchymal transition (EMT)-induced enrichment of PD-L1.^26^ Conversely, phosphorylation of PD-L1 S195 by AMP-activated protein kinase (AMPK) induced abnormal glycosylation of PD-L1 and blocked its ER to Golgi transportation, resulting in ER-associated degradation (ERAD).^27^ In the cases of some cancer cells, the glycan modification rendered PD-L1 undetectable by conventional antibodies, which led to misinterpretation of PD-L1 surface level.^28^ Removal of N-glycosylation led to more faithful detection of PD-L1 surface level.^28^ This finding reflects the fact that glycosylation patterns of PD-L1 can vary in different cancer cells, which might be due to their different microenvironments,^29^ and some patterns prevent binding of conventional antibodies.

Surface PD-L1 undergoes constant internalization, followed by recycling or degradation (Fig. 1b). A chaperone protein, CKLF-like MARVEL transmembrane domain containing 6 (CMTM6) that belongs to a family of eight MARVEL domain-containing proteins with previously unknown function, regulates PD-L1 recycling. CMTM6 associates with PD-L1 at both the plasma membrane and endosomes to facilitate recycling and also inhibits ubiquitination and degradation by the lysosome. Tumor cells with CMTM6 deficiency showed reduced PD-L1 recycling and surface level, leading to less suppression of T cell activity.^30^ CMTM4, an analog of CMTM6, also has a similar function.^31^ How CMTM6/4 supports PD-L1 recycling remains undefined. Multiple proteins were identified to regulate lysosome degradation of PD-L1. HIP1R carries a lysosome sorting motif, and its binding with PD-L1 targets PD-L1 to the lysosome with the help of the AP complex and ALIX/ESCRT.^32^ It was suggested that STUB1 E3 ubiquitin ligase can mediate PD-L1 degradation in the lysosome.^30,31^ Whether STUB1 cooperates with HIP1R is still unknown. According to the work from several groups, the proteasome is also involved in PD-L1 degradation. Cullin 3-SPOP, β-TrCP, and HRD1 E3 ligases were reported to regulate PD-L1 ubiquitination and proteasomal degradation, and they seem to regulate PD-L1 degradation in different contexts. During cell cycling, PD-L1 surface level showed evident fluctuations, peaking in M and early G1 phases and quickly declining in late G1 and S phases. This fluctuation was regulated by the cyclin D-CDK4-SPOP-FZR1 signaling pathway. CDK4 phosphorylated and stabilized SPOP, an adaptor protein in the Cullin 3-based E3 ubiquitin ligase complex, to mediate PD-L1 polyubiquitination and degradation by the proteasome.^33^ Interestingly, glycosylation can directly affect PD-L1 ubiquitination and degradation through β-TrCP and HRD1. When PD-L1 was not glycosylated, it can be phosphorylated by glycogen synthase kinase 3β (GSK3β) at T180 and S184 and recruit β-TrCP to mediate PD-L1 ubiquitination and degradation.^34^ On the other hand, S195 phosphorylation caused abnormal glycosylation of PD-L1, which resulted in recruitment of HRD1 to trigger ER-associated degradation.^27^

There are also sophisticated mechanisms antagonizing PD-L1 ubiquitination and degradation. COP9 signalosome 5 (CSN5) was reported to deubiquitinate PD-L1, thereby inhibiting PD-L1 degradation. NF-κB pathway activated by TNF-α induced CSN5 expression to stabilize PD-L1 expression in cancer cells.^35^ Palmitoylation of PD-L1 at C272 by DHHC3 blocked mono-ubiquitination of PD-L1 and the subsequent ESCRT-mediated trafficking to multivesicular bodies (MVB), resulting in suppression of PD-L1 lysosomal degradation.^36^

### CTLA-4 regulation

Differing from PD-1 whose primary location is at the plasma membrane, CTLA-4 is mainly localized in intracellular compartments. Upon T cell activation, CTLA-4 translocates to the cell surface to mediate its inhibitory function.^37^ T cell receptor-interacting molecule (TRIM) is required for CTLA-4 trafficking from the *trans* Golgi network (TGN) to the cell surface. TRIM knockdown led to retention of CTLA-4 in the TGN.^38^ A subsequent study showed that a CTLA-4/TRIM/LAX/Rab8 complex was essential for this trafficking pathway.^39^ Phospholipase D (PLD)- and ADP ribosylation factor-1 (ARF1)-dependent exocytosis was also reported to trigger the trafficking of CTLA-4 to the cell surface.^40^

Surface CTLA-4 molecules are rapidly internalized to maintain relatively low surface levels (Fig. 1c). The clathrin-associated adaptor complex AP-2 binds to the YVKM motif in the CTLA-4 cytoplasmic domain to mediate internalization, which can be prevented by YVKM phosphorylation^41^. However, another study showed that YVKM-mediated CTLA-4 internalization was not impaired during T cell activation, thus suggesting that YVKM phosphorylation might not regulate CTLA-4 internalization directly.^42^ Another clathrin adaptor complex, AP-1, also binds to the YVKM motif, but differs in that it shuttles CTLA-4 from the TGN to lysosomes for degradation.^43^ Additionally, the internalization rate of CTLA-4 is also regulated by N-glycosylation. Vitamin D3 treatment enhanced N-acetylglucosaminyltransferase I (Mgat1) expression and N-glycan branching, leading to reduced internalization and increased surface level of CTLA-4 in T cells.^44^ N-glycosylation is also essential for CTLA-4 surface delivery. A T17A polymorphism in the signal peptide led to insufficient glycosylation and lower CTLA-4 surface level.^45^ TCR signaling was shown to increase hexosamine metabolism and N-glycan-branching pathway, therefore increasing CTLA-4 glycosylation and surface expression.^46^ Internalized CTLA-4 in endosomes can be recycled back to the cell surface.^42^ LPS responsive beige-like anchor protein (LRBA) co-localizes with CTLA-4 in recycling endosomes to assist its recycling. LRBA mutation in human patients reduces CTLA-4 levels in regulatory and conventional T cells, which leads to the phenotypes of autoimmunity, lymphoproliferation, and humoral immune deficiency.^47^

## Checkpoint signaling mechanisms

**Fig. 2:**
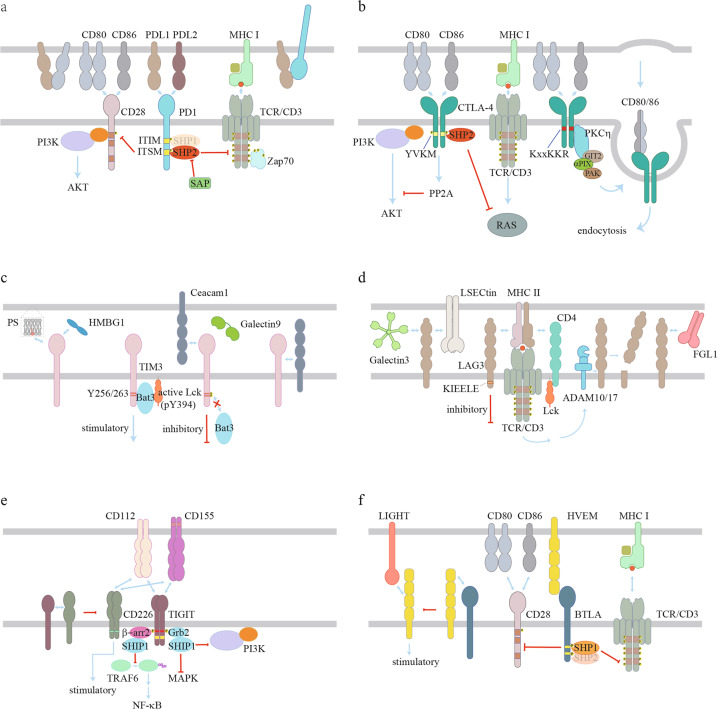
Ligand binding and signal transduction of immune checkpoint receptors. **a** PD-L1 and PD-L2 are ligands for PD-1. PD-1 recruits protein tyrosine phosphatase SHP2/SHP1 via phosphorylated ITSM/ITIM, which in turn inhibits both TCR and CD28 signaling. SAP inhibits SHP2 activity to suppress PD-1 signaling. Both PD-1 and CD80 interact with PD-L1 in *cis* to restrict its *trans* ligation of PD-1. **b** CTLA-4 competes with CD28 on binding with CD80/86 binding to inhibit CD28 signaling. The phosphorylated YVKM motif of CTLA-4 recruits SHP2 to inhibit RAS. CTLA-4 also inhibits AKT activity through PP2A. CTLA-4 in Tregs reduces CD80/86 on APCs by *trans*-endocytosis, which requires KxxKKR motif and PKCη. **c** TIM3 expresses in both T cells and innate immune cells. Four known ligands have been identified: Ceacam1, Galectin9, HMBG1, and PS. Galectin9 binds to glycosylated IgV domain of TIM3 in T cells. Ceacam1 exhibits both *cis* and trans interactions. *Cis* interaction of Ceacam1 with TIM3 is essential for TIM3 surface expression in T cells. In the absence of ligands, Bat3 binds to unphosphorylated Y256/263 in TIM3 cytoplasmic domain and recruits active Lck to deliver stimulatory signal in T cells. Interaction with Galectin9/Ceacam1 leads to phosphorylation of TIM3 Y256/263 and the subsequent abolishment of Bat3 binding, thus converting TIM3 from a stimulatory to an inhibitory molecule. TIM3 in DCs binds with PS and HMBG1 to regulate innate immunity. **d** LAG3 binds to MHC-II to inhibit CD4-dependent T cell function with its cytoplasmic domain. TME-derived Galectin3, LSECtin and FGL1 bind with LAG3 to inhibit T cell function, which requires the KIEELE motif in the LAG3 cytoplasmic domain. TCR signaling upregulates activity of ADAM10 and ADAM17, which cleave LAG3 at the extracellular domain to abolish its suppression of T cell signaling. **e** TIGIT and CD226 bind to the same ligands, CD112 and CD155. CD226 is a co-stimulatory receptor whereas TIGIT is a co-inhibitory receptor. TIGIT binds with CD112/CD155 with higher affinity than CD226 and inhibits the PI3K, MAPK and NF-κB pathways by recruiting SHIP1. **f** BTLA interacts with HVEM both in *trans* and *cis*. The *cis* interaction between BTLA and HVEM inhibits the *trans*-ligation of HVEM by LIGHT and thus inhibits HVEM stimulatory signaling triggered by LIGHT binding. ITIM and ITSM in BTLA recruit SHP1/SHP2 to inhibit both TCR and CD28 signaling.

The suppressive functions of immune checkpoints usually depend on ligand-induced signaling. Here we summarize ligand interactions and signaling mechanisms of several well studied immune checkpoints (Fig. 2).

### PD-1 signaling

PD-1 signaling can be triggered by its engagement with the ligands PD-L1 and PD-L2. Conventionally, PD-L1 or PD-L2 expressed on the surface of antigen presenting cells or tumor cells engages in *trans* interactions with PD-1 expressed on the surface of T cells to induce inhibitory signaling.^48–50^ Tumor cells can also secrete PD-L1-containing extracellular vesicles, mainly in the form of exosomes, to activate PD-1 pathway. These exosomal PD-L1 molecules primarily suppress T cell activity in the draining lymph node. Melanoma patients who were not responsive to anti-PD-1 therapy showed higher levels of exosomal PD-L1 than responders.^51,52^ Recent studies show that the PD-1/PD-L1 interaction can also occur in *cis*. The co-expression and interaction between PD-L1 and PD-1 on APCs prevented the *trans*-ligation of PD-1, thus decreasing the inhibitory function of T cell PD-1.^53^ In addition to PD-1, PD-L1 can interact with CD80 *in cis*,^54–56^ which can disrupt PD-L1/PD-1 and CD80-CTLA-4 interactions but preserve the ability of CD80 to activate CD28 signaling.^56,57^ Thus, the *cis* PD-L1-CD80 interaction plays positive roles in antitumor immunity by inhibiting PD-1 and CTLA-4 function.

After ligand engagement, PD-1 becomes phosphorylated to deliver inhibitory function (Fig. 2a). An ITIM and an ITSM are present in its cytoplasmic domain. The phosphorylated ITSM might be more important, as mutation of tyrosine in ITSM but not ITIM, significantly abrogated the inhibitory function of PD-1.^58–60^ Phosphorylated ITSM primarily recruits SHP2 to dephosphorylate key signaling molecules to downmodulate activation level.^61–63^ Although ITIM is usually considered to be dispensable for PD-1 inhibitory function, recent studies suggested that ITIM plays a role in converting SHP2 from inactive to active conformation.^64,65^ Signaling lymphocytic activation molecule-associated protein (SAP) was shown to block SHP2 interaction with its substrate and thus could inhibit PD-1 signaling.^64^ Although SHP2 is essential for PD-1 inhibitory signaling in most cases, *Shp2*-deficient T cells can still acquire dysfunctional features and respond to α-PD-1 treatment in vivo, suggesting an alternative signaling mechanism.^62^ Several groups reported that SHP1 can also be recruited by phosphorylated PD-1 ITSM.^59,66^ A recent study applied mass spectrometry to quantitatively illustrate PD-1 signalosome assembly.^63^ They found that SHP2 was predominantly recruited by PD-1, while SHP1 serves as a reserve to compensate for loss of SHP2 or in the event that SHP2 becomes limited, a likely scenario in chronic/dormant T cells with typically high expression of PD-1.^63^ Intriguingly, PD-1 still potently inhibited proliferation and cytokine production of primary T cells with *Shp1/2* double knockout,^67^ suggesting an unknown mechanism underlying PD-1 inhibitory function.

It has been shown that PD-1 inhibits both antigen and co-stimulatory signaling.^58,60,61,63,66,68^ In activated T cells, PD-1 translocates to the immunological synapse and therefore is in close proximity to both TCR and CD28. A biochemical study showed that SHP2 had significant preference for CD28 over TCR.^61^ Indeed, the downstream PI3K-AKT pathway of CD28 was inhibited by PD-1 in an ITSM-dependent and ITIM-independent manner.^58^ However, PD-1 signaling was also shown to inhibit phosphorylation of TCR and downstream signaling molecules like ZAP70.^60,66^ A transcriptional analysis of PD-1-modulated gene expression during early T cell activation showed that PD-1 mainly suppressed genes induced by strong TCR signaling.^69^ It is likely that SHP2 recruited by phosphorylated PD-1 ITSM prefers CD28 over TCR but can still inhibit TCR signaling. In addition to its inhibitory roles in T cell signaling, SHP2 was also reported to activate TCR signaling by reversing CSK-mediated inhibitory phosphorylation of LCK. Evidence suggest that sequestration of SHP2 by phosphorylated PD-1 prevent SHP2 from stimulating LCK activity, thus contributing to suppression of T cell signaling.^70^

### CTLA-4 signaling

Compared to CD28, CTLA-4 binds to CD80/86 with higher affinity,^71^ thus inhibiting co-stimulation by ligand competition. In addition, CTLA-4-expressing T cells can reduce CD80/86 expression on APCs by *trans*-endocytosis, resulting in decreased CD28 signaling.^72^ For example, regulatory T (Treg) cells with constitutive CTLA-4 expression can mediate CD80/86 downregulation on dendritic cells (DCs) through this *trans*-endocytosis process, which is required for the suppressive function of Treg cells.^73^ As mentioned above, a *cis*-CD80/PD-L1 heterodimer on APCs protects CD80 from CTLA-4-mediated *trans*-endocytosis.^56^ Although the *cis* interaction between PD-L1 and CD80 disrupts inhibitory function of both PD-1 and CTLA-4, tumor cells often have low CD80 expression such that this protective mechanism might not be effective.

Upon T cell activation, CTLA-4 translocates to the cell surface and clusters into the immune synapse.^74,75^ The tyrosine in the YVKM motif of CTLA-4 can be phosphorylated by Src family kinases or other kinases, such as Jak2 and Rlk^76–78^ (Fig. 2b). Tyrosine phosphorylation prevents the interaction between CTLA-4 with AP-2, therefore maintaining CTLA-4 on the cell surface to deliver inhibitory signaling.^41^ On the other hand, the YVKM motif might also recruit SHP2 to repress T cell activation.^79^ In addition, although the direct recruitment of PP2A by CTLA-4 is still under debate,^75,80^ the inhibition of AKT activity by CTLA-4 is PP2A-dependent.^58^ In Treg cells, PKCη was recruited to the immune synapse by CTLA-4. CTLA-4/PKCη further recruited GIT2-αPIX-PAK complex, which facilitated Treg-APC interaction and was required for contact-dependent suppression by Treg cells.^81^ In addition to cytoplasmic tail-mediated inhibition of the T cell response, CTLA-4 is also thought to inhibit T cell signaling in extrinsic manners. For example, CTLA-4 reduces CD80/86 expression on APCs by either trans-endocytosis as mentioned above or by inducing tumor growth factor β (TGFβ) that in turn downregulates CD80/86.^82^ CTLA-4 also induces indoleamine 2,3-dioxygenase (IDO) expression in DCs via ligation of CD80/86, resulting in tryptophan depletion and T cell suppression.^83^

### TIM3 signaling

Four ligands have been reported for TIM3, namely C-type lectin galectin9 (Galectin9), carcinoembryonic antigen cell adhesion molecule 1 (Ceacam1), high-mobility group box 1 (HMGB1), and a non-protein ligand phosphatidylserine (PS) (Fig. 2c). Galectin9 is a soluble protein with two carbohydrate recognition domains. The binding of Galectin9 to TIM3 required glycosylated IgV domain of TIM3.^84^ Ceacam1 binds to TIM3 both in *cis* and *trans*. The co-expression and *cis* interaction with Ceacam1 were essential for TIM3 glycosylation and surface expression, while *trans* interaction mediated inhibition of effector T cell function.^85^ The other two ligands mainly regulate innate immune response. HMGB1 is a non-histone chromatin-associated protein that can be secreted to the TME. HMGB1 bound to TIM3 on tumor-associated DCs to suppress the recruitment of released nucleic acid from dying tumor cells to the endosome of DC, thus inhibiting nucleic acid-induced innate immune response.^86^ Furthermore, TIM3 acts on efferocytosis-recognized apoptotic cells via direct PS binding to regulate efferocytosis in DCs. TIM3 antibody inhibited engulfment of apoptotic cells by CD8^+^ DC, thereby reducing antigen cross-presentation.^87,88^

TIM3 signaling remains controversial, as different groups have reported opposite effects of TIM3 in T cell effector function. In an murine acute lymphocytic choriomeningitis virus (LCMV) infection model, TIM3 expression promoted short-lived effector T cell development, accompanied with enhanced AKT/mTOR signaling.^89^ Another work showed that TIM3 interacted with multiple proximal TCR signaling molecules in the immune synapse, with TIM3 blockade enhancing stable synapse formation between TIM3^high^ CD8 T cells and target cells.^90^ TIM3 contains five conserved tyrosine residues in its cytoplasmic domain, among which Y265 (Y256 in mouse) and Y272 (Y263 in mouse) can be phosphorylated by Src family kinases^91^ or interleukin-2-inducible T cell kinase (ITK).^92^ Upon phosphorylation, these tyrosine residues can recruit p85 to promote NFAT activation.^91^ Bat3 acts as an inhibitor of TIM3-induced cell death and exhaustion in Th1 cells. Upon binding to unphosphorylated TIM3 cytoplasmic domain, Bat3 specifically recruits the catalytically active form of Lck to promote TCR signaling. TIM3 binding with antibody or ligand causes dissociation of Bat3, likely through the phosphorylation of Y265 and Y272, and reverses the inhibitory effects of Bat3 on TIM3 function.^85,93^ It is therefore possible that while TIM3 itself might act as an inhibitory receptor, its association with Bat3 converts it to stimulatory in some contexts.

### LAG3 signaling

LAG3 is identified as a ligand of MHC-II with higher affinity than CD4^94,95^ and thus might inhibit CD4^+^ T cell activation by preventing CD4-MHC-II interaction. However, other studies showed that the inhibitory function of LAG3 is independent of CD4 competition but rather dependent on its cytoplasmic domain to deliver inhibitory signaling.^96,97^ Nevertheless, the inhibitory function of LAG3 in CD8^+^ T cells does not involve MHC-II, suggesting that other ligands might exist for LAG3. Indeed, LSECtin and Gelectin-3 bind to LAG3 and suppress T cell function in the TME^98,99^ (Fig. 2d). LAG3 is a glycoprotein with four potential N-linked glycosylation sites in the extracellular domain.^94,100^ Considering both LSECtin and Gelectin-3 are carbohydrate-binding proteins, their LAG3 binding may be dependent on the glycosylation of LAG3. Recently, fibrinogen-like protein 1 (FGL1) was identified as a new ligand for LAG3. Normally, FGL1 is released into blood at low levels from the liver. However, upregulated FGL1 is detected in several human cancers. Blocking the interaction between FGL1 and LAG3 can enhance the antitumor function of T cells.^101^ It is interesting to point out that LAG3 is also expressed in Tregs to inhibit proliferation and function.^102^ Meanwhile, ligation of MHC-II on APCs by Treg-expressed LAG3 also suppresses APCs function.^103,104^ Therefore, the roles of LAG3 are complex and using LAG3 blockade for cancer immunotherapy needs to be carefully studied to provide clinical benefits.

Knowledge on the signal transduction of LAG3 is still limited. Crosslinking of CD3 and LAG3 inhibited T cell proliferation and cytokine production, which may be caused by impairing proximal TCR signaling as a reduction of calcium influx was also observed.^105^ The cytoplasmic domain of LAG3 contains three conserved regions in both human and mouse, a serine phosphorylation site, KIEELE motif, and multiple EP repeats. The KIEELE sequence is essential for the inhibitory function of LAG3 in CD4^+^ T cells.^96^ LAG3 function can be antagonized by TCR signaling through two transmembrane metalloproteases (A Disintegrin And Metalloproteinase domain-containing protein 10 and 17 (ADAM10 and ADAM17)) that can cleave LAG3. TCR signaling upregulates the cleavage activity of ADAM10 and ADAM17 by distinct mechanisms, which in turns allows efficient T cell proliferation and function.^106^

### TIGIT signaling

CD155 (PVR) and CD112 (PVRL2) are two ligands for TIGIT, with CD155 having a higher affinity than CD122.^107^ *Trans* ligation of TIGIT not only delivers inhibitory signals in T and natural killer (NK) cells via TIGIT signaling,^108,109^ but also suppresses T cell function by enhancing IL-10 production of DCs via reverse CD155 signaling.^107^ CD226, a co-stimulatory receptor, shares the same ligands with TIGIT.^110^ However, the affinity of TIGIT to its ligands is higher than CD226, and therefore TIGIT can suppress CD226-mediated co-stimulation via ligand competition.^111^ Interestingly, TIGIT can also directly bind to CD226 in *cis* to disrupt its homodimer formation and co-stimulatory function.^112^

The signal transduction of TIGIT is mainly studied in NK cells. TIGIT cytoplasmic domain contains an ITIM motif and an immunoglobulin tail tyrosine (ITT)-like motif (Fig. 2e). Different studies show that tyrosine phosphorylation in either ITIM motif or ITT-like motif is essential for inhibitory function of TIGIT in human NK cells.^108,113,114^ However, in murine NK cells these two motifs seem to be redundant.^115^ The ITT-like domain is reported to recruit SHIP1 through two adaptor proteins Grb2 and β-arrestin2. The Grb2-recruited SHIP1 predominantly inhibits PI3K and MAPK signaling,^113^ while β-arrestin2-recruited SHIP1 mainly impairs TRAF6 to abolish NF-κB activation.^114^ However, downstream signals of ITIM motif in TIGIT are still unclear.

### BTLA signaling

BTLA and CD160 share the same ligand, herpesvirus entry mediator (HVEM), to suppress T cell function.^116–118^ However, HVEM itself delivers co-stimulatory signal when engaged with TNF superfamily member LIGHT or BTLA/CD160.^119–121^ BTLA/CD160 and LIGHT bind to different sites of HVEM with BTLA/CD160 interacting with the cysteine-rich domain 1 (CRD1) region. CRD1 truncation of HVEM however does not affect LIGHT binding.^117,122^ Interestingly, soluble LIGHT enhances BTLA/HVEM interaction, while membrane-associated LIGHT purportedly displaces BTLA due to its higher affinity for HVEM.^123^ *Cis* interaction occurs when BTLA and HVEM are co-expressed, which prevents HVEM from being activated by *trans* ligation.^124^

BTLA contains ITIM and ITSM motifs as well as a Grb2 recognition motif in its cytoplasmic domain.^116^ Both tyrosine residues in ITIM and ITSM can be phosphorylated and recruit SHP1/SHP2 to inhibit T cell function after ligation^116,117,125^ (Fig. 2f). Further comparison between BTLA and PD-1 signaling showed that in opposition to PD-1 which recruits the weaker phosphatase SHP2, BTLA prefers to recruit the more potent phosphatase SHP1, to more effectively inhibit both TCR and CD28 signaling.^63,126^ In addition, BTLA on T follicular helper (Tfh) cells recruits SHP1 to the immune synapse when engaged with HVEM expressed on B cell surface, which inhibits TCR signaling and restrains CD40L to inhibit B cell proliferation.^127^

## Therapeutic strategies targeting immune checkpoint expression

Immune checkpoint blockade therapy using antibodies to block receptor-ligand interactions has gained ground and been approved for clinical use. However, the overall response rate for these blockade antibodies is still low.^5,6,10^ Given that the inhibitory function of immune checkpoints is critically regulated by their surface expression and signal transduction, targeting these pathways can provide novel strategies for immunotherapy (Table 1).

**Table 1:** New therapeutic strategies targeting immune checkpoints.

Drug candidate	Function	Mechanism	Reference
2F-Fuc	Downregulate PD-1	Core fucosylation is required for PD-1 surface expression. 2F-Fuc inhibits fucosylation to reduce PD-1 surface levels on activated T cells.	^18^
IL-2		IL-2 induces Fbxo38 expression through STAT5, which in turn mediates PD-1 ubiquitination and degradation.	^22^
Gefitinib	Downregulate PD-L1	GSK3β interacts with PD-L1 and induces degradation of PD-L1 by β-TrCP. EGF signaling inactivates GSK3β to stabalize PD-L1 in basal-like breast cancer. Gefitinib inhibits EGF signaling to destabilize PD-L1.	^34^
Metformin		Metformin activates AMPK to phosphorylate PD-L1 at S195, which leads to abnormal PD-L1 glycosylation and ERAD-mediated PD-L1 degradation.	^27^
Etoposide		EMT induces expression of N-glycosyltransferase STT3, which is required for PD-L1 glycosylation and stabilization. Etoposide inhibits EMT/β-catenin/STT3/PDL1 axis to downregulate PD-L1.	^26^
Peptide (PD-LYSO)		PD-LYSO consists of PD-L1-binding sequence and lysosome-sorting signal of HIP1R to target PD-L1 for lysosomal degradation.	^32^
Curcumin		CSN5 stabilizes PD-L1 via deubiquitination. Curcumin inhibits enzyme activity of CSN5 to destabilize PD-L1.	^35^
2-bromopalmitate		Inhibition of PD-L1 palmitoylation abolishes its suppression of PD-L1 mono-ubiquitination and degradation.	^36^
Palbociclib	Upregulate PD-L1	CDK4/6 inhibitor palbociclib inhibits Cyclin D1-CDK4-mediated phosphorylation and stabilization of SPOP, an E3 ligase for PD-L1, causing upregulation of PD-L1 in cancer cells.	^33^
pH-sensitive anti-CTLA-4 antibody	Abrogate irAE	pH-sensitive anti-CTLA-4 antibody prevents antibody-triggered lysosomal degradation of CTLA-4 and attenuates irAE.	^130^

Many pioneering studies have explored the possibility of targeting checkpoint glycosylation and ubiquitination/degradation pathways. These experiments were performed in different systems and here we list them together to highlight the translational potential of this new approach: (1) Targeting checkpoint glycosylation. Proper glycosylation is required for stable surface expression of checkpoint. Treating T cells with fucosylation inhibitor 2-fluoro-L-fucose (2F-Fuc), reduces the fucosylation and surface level of PD-1. 2F-Fuc-treated cytotoxic T lymphocytes (CTLs) show enhanced antitumor immunity during adoptive cell transfer (ACT) therapy.^18^ PD-L1 glycosylation is regulated by AMPK and EMT. Metformin, a widely used drug for type 2 diabetes, activates AMPK to induce abnormal glycosylation and degradation of PD-L1. Etoposide, a chemotherapy medication used to treat various cancers, inhibits EMT-induced PD-L1 glycosylation to destabilize surface PD-L1. Downregulation of surface PD-L1 in tumor cells by metformin and etoposide enhances the efficacy of anti-CTLA-4 and anti-TIM therapies.^26,27^ PD-L1 glycosylation is also regulated by EGFR signaling. Gefitinib, an EGFR inhibitor that is used as a treatment in many cancers, also inhibits PD-L1 glycosylation and in turn promotes GSK3β-mediated ubiquitination and degradation, which results in the enhancement of efficacy of anti-PD-1 therapy.^34^ (2) Targeting checkpoint ubiquitination/degradation. Promoting degradation of checkpoints appears to be an interesting direction. IL-2, an FDA-approved drug for metastatic melanoma and renal cancer, upregulates FBXO38-mediated PD-1 ubiquitination/degradation. This likely represents one of the several working mechanisms of IL-2 in treating cancer.^22^ A rationally-designed peptide PD-LYSO, containing a PD-L1-binding sequence and a lysosomal-sorting signal sequence from HIP1R, can target PD-L1 for lysosomal degradation.^32^ Curcumin inhibits deubiquitination activity of CSN5 to destabilize PD-L1 and benefits anti-CTLA-4 therapy.^35^ As mentioned above, PD-L1 palmitoylation can suppress mono-ubiquitination and degradation to stabilize surface expression. 2-bromopalmitate inhibits PD-L1 palmitoylation to reduce PD-L1 surface level and in turn promotes antitumor immunity in the murine MC38 tumor model.^36^ On the other hand, upregulating PD-L1 surface level has also been shown to be beneficial under certain circumstances. The CDK4/6 inhibitor palbociclib inhibits cyclin D-CDK4-SPOP-FZR1 pathway-mediated PD-L1 ubiquitination and degradation, which increases PD-L1 level and sensitizes CT26-implanted tumor to anti-PD-1 therapy.^33^ Lastly, CTLA-4 blockade antibodies used for cancer immunotherapy often induce severe immunotherapy-related adverse effects (irAEs).^128,129^ A recent study showed irAE-prone CTLA-4 blocking antibodies induced lysosomal degradation of CTLA-4, while non-irAE-prone antibodies allowed CTLA-4 recycling in an LBRA-dependent manner. Increasing pH sensitivity of irAE-prone anti-CTLA-4 antibodies can prevent antibody-triggered lysosomal degradation of CTLA-4 and attenuates irAE.^130^

## Perspective

Inhibitory functions of immune checkpoints are tightly regulated by surface expression level, receptor-ligand interactions, and intracellular signal transduction. Current immune checkpoint blockade therapies are designed to target the receptor-ligand interaction. In addition to this successful approach, recent studies have shown that modulating surface expression and intracellular signaling might represent other exciting avenues to reinvigorate antitumor immunity. Despite much exciting progress made in the field, several topics remain to be addressed by future research to pave the way for next-generation immunotherapies: (1) Post-translational modifications (PTMs) of immune checkpoints. Current studies highlight the importance of glycosylation, lipid modification, and ubiquitination in checkpoint function. However, our understanding of checkpoint PTMs is still very limited. Advanced mass spectrometry techniques will be needed to systematically investigate checkpoint modifications. (2) Turn-over processes of immune checkpoints. As membrane proteins, surface expression levels of immune checkpoints are controlled by several cell biology processes, including surface delivery, internalization, recycling and degradation. These processes are poorly understood thus far and identifying the key regulatory proteins involved is therefore warranted. (3) Intracellular signaling mechanisms of immune checkpoints. Most checkpoints require tyrosine phosphorylation to activate inhibitory signaling, but the phosphorylation processes are not well studied. Moreover, effector molecules recruited upon checkpoint phosphorylation are also not well characterized. Indeed, it appears that different checkpoints prefer distinct effector molecules to execute their functions. For example, PD-1 primarily recruits SHP2 while BTLA primarily recruits SHP1. The underlying significance of these distinct specificities is unclear. Whether SHP2 and SHP1 play distinct roles in immune suppression is also not fully understood. More experiments therefore need to be performed in the future to fill in these gaps. (4) Context-dependent biology of immune checkpoints. Recent findings strongly suggest that immune checkpoints are subject to specific regulatory mechanisms and exhibit distinct functions in different immune and cancer cell contexts. The tumor microenvironment is packed with various cell types in addition to cancer cells, including T cells, B cells, macrophages, neutrophils, DCs, myeloid-derived suppressor cells, NK cells and cancer-associated fibroblasts. It will be of great interest to both basic and translational researchers to have a more complete picture of checkpoint biology of these different cell types within the TME. For example, the roles of PD-1 in effector, regulatory and memory T cells are complex and multifactorial. Indeed, PD-1 blockade can cause Treg overaction and lead to immune suppression instead of immune reinvigoration, as reflected by the hyperprogressive disease observed in some melanoma patients receiving PD-1 blockade therapy.^131^

In summary, immunotherapies based on checkpoint biology represent a bright future for the treatment of cancer. Expanding our understanding of immune checkpoint biology will improve the efficacy of current checkpoint blockade therapies and also inform the generation of novel immunotherapy approaches for translation into the clinic.
